# Aims, design and preliminary findings of the Hellenic National Nutrition and Health Survey (HNNHS)

**DOI:** 10.1186/s12874-018-0655-y

**Published:** 2019-02-20

**Authors:** Emmanuella Magriplis, Ioannis Dimakopoulos, Dimitra Karageorgou, Anastasia-Vasiliki Mitsopoulou, Ioanna Bakogianni, Renata Micha, George Michas, Triantafyllia Ntouroupi, Sophia-Maria Tsaniklidou, Kostantina Argyri, George Danezis, Constantinos Georgiou, Demosthenes B. Panagiotakos, Antonis Zampelas, HNNHS Contributors, Evangelia Fappa, Eleni-Maria Theodoraki, Eirini Trichia, Theodora-Eirini Sialvera, Aggeliki Varytimiadi, Eleni Spyreli, Michalis Chourdakis, Antonis Koutelidakis, George Karlis, Stauroula Zacharia, Anna Papageorgiou, HNNHS Advisory Committee, George P. Chrousos, Georgios Dedoussis, George Dimitriadis, Ioannis Yannis Manios, Eleftheria Roma

**Affiliations:** 1 Department of Food Science and Human Nutrition, Agricultural University of Athens, Iera odos 75, 118 55 Athens, Greece; 2 Department of Nutrition and Dietetics, School of Health Science and Education Harokopio University, Athens, Eleftheriou Venizelou 70, 176 76 Athens, Greece; 3 Present Address: Friedman School of Nutrition Science and Policy, Tufts University, Boston, USA; 4 Present Address: Department of Cardiology, “Elpis” General Hospital of Athens, Athens, Greece

**Keywords:** Diet, Public health, Nutrition survey/ methods, Cross-sectional study, Greece

## Abstract

**Background:**

The aim of the Hellenic National Nutrition and Health Survey was to assess nutritional intake, health status and various behaviors in a representative sample of the Greek population.

**Methods:**

Data collection took place from 01.09.2013 to 31.05.2015. Random stratified sampling was performed by (a) geographical density criteria of Greece (7 regions), (b) age group of the reference population (< 19, 20–64 and > 65 years) and (c) gender distribution. The final population enrolled included (throughout Greece), 4574 individuals (42.5% men; 57.5% women of who 47.2% were from Athens metropolitan area, 18.5% from Central Macedonia, and the remaining 34% almost equally scattered throughout the country (p for the comparisons with official statistics by region, age group and sex > 0.7). Questionnaires developed were based on extensive review of the literature, following a validation procedure when necessary.

**Results:**

Preliminary analyses revealed that 32% of the adult population were overweight and 15.5% were obese, with significant gender differences in total and per age group (*p* < 0.001, for all). The majority of the adult population reported being active smokers (50.4%) or regular alcohol consumers (72.4%); with significant gender differences (*p* < 0.001, for all). Prevalence of hyperlipidemia was 16.7%, cardiovascular disease 13.9%, hypertension 13.3%, thyroid disease 13.8%, and Diabetes Mellitus 3.6%. Significant gender and age group differences were found in various diseases.

**Conclusions:**

Study’s preliminary results provide valuable information about the Hellenic population’s health. Findings from this survey could be used to detect disease risk factors for public health prevention policies and programs.

**Electronic supplementary material:**

The online version of this article (10.1186/s12874-018-0655-y) contains supplementary material, which is available to authorized users.

## Background

The evaluation of current population’s mental and physical health and the identification of the most important modifiable risk factors for disease prevention and treatment is mandatory in assuring a healthy and productive population [1–5].

During the last few decades, a pharmacological approach for public health promotion was widely used, hence focusing mostly on disease treatment rather than prevention. This approach allowed increased prevalence of various health risk factors, and led to an increase in health care costs, and a decrease in gross production. World data have shown that 8 out of the 20 main causes of morbidity and mortality are due to unhealthy nutrition [1, 4, 5]. Recent findings showed that the three leading factors of global disease burden were high blood pressure, smoking, and high alcohol consumption [4, 5], however when globally assessed their geographical variations on the magnitude of their effect of these risks varied with alcohol being the leading risk factor in Eastern Europe and high blood pressure in central Europe, for example. Additionally, overweight and obesity, physical inactivity and other modifiable risk factors (dietary fats) represent substantial risk factors too, with their risk burden varying on diseases by gender, and dietary fats by age (children, adults, elderly) [4, 5]. More specifically, older ages had a higher consumption of fish oils, while younger individuals had a higher trans-fat intake [5].

Therefore, well-designed country specific studies are necessary for the assessment and evaluation of major modifiable risk factors in different geographic regions, which will enable a focused (per region’s needs) promotion of public health. Additionally, data should also focus in gender and age specific differences.

Efficiently performed well-designed nationally representative cross-sectional studies have adequately evaluated the population’s health and nutrition habits. Examples from such programs include the National Health and Nutrition Examination Survey (NHANES) and the National Diet and Nutrition Survey (NDNS). During the last several decades, findings from NHANES have been used in the United States of America (USA) for status and the development of health policies to safeguard public health, including policies for prevention of lead poisoning and folic acid deficiency through compulsory food fortification [6, 7].

The present Hellenic National Nutrition and Health Survey (HNNHS) is the first national cross-sectional study that takes place in Greece, which encompasses a representative sample of all ages, and following standards established by NHANES (USA) and NDNS (United Kingdom).

The aim of the HNNHS was to assess nutritional intake, health status and various behaviors in the Greek population, which could be used to help promote public health through the design and implementation of related policies and intervention programs. In the present paper, the aims, the design and some preliminary findings of the HNNHS are explained below in detail.

## Methods

### Study design

This is a cross-sectional observational survey. Responders’ selection was performed with a random stratified design based on the 2011 census data. Stratification was made according to (a) geographical density criteria by Greek region (7 regions), as provided by the Hellenic Statistical Authority, (b) age group of the reference population (< 19, 20–65 and > 65 years) and (c) gender distribution. A random selection of more than one individual per household was possible but no more than one individual from the same age group could be enrolled in the study. If households had children < 6 years of age, one (if more were present) was randomly selected to be included in the study, upon consent. The sample required to accurately evaluate measures of effect for common risk factors and prevalence of chronic diseases (a priori estimated to equal to 1.2), at 0.05 level (alpha) was 3634 individuals, to achieve a statistical power equal to 85%. To maintain 85% power in the estimation of prevalence rates of chronic diseases or morbidities equal to 15%, with 1 standard deviation (SD) of the referent population (*N* = 11,000,000), at 0.05 significance, a sample size of 4658 was needed.

### Sample

Invitations were sent to approximately 6000 individuals (anticipating a 70–75% response rate) in to achieve the required sample size, based on a feasibility and volunteer basis in all Greek regions, by the study’s investigators from 01.09.2013 to 31.05.2015**.** A total 4574 (42,5% men and 57.5% women) finally agreed to participate. The sample was distributed throughout Greece, with 47.2% of it residing in the Athens Metropolitan area, 18.5% in the region of Central Macedonia, whereas the rest was almost equally scattered throughout the country (Table 1; p for the comparisons with Official statistics by region, age group and sex > 0.7). Post-hoc assessment, accounting for large population (*N* > 10,000) resulted in a 92% study power, for an effect size of 1.2 (OR = 1.2). When the 15% probability of chronic disease was accounted for, the power was reduced to 84%.

**Table 1:** Distribution of the sample within Greece

Prefecture	N	%
Attica	2160	47.2
Central Macedonia	844	18.5
Epirus	59	1.3
Eastern Macedonia, Thrace	193	4.2
Peloponnese	144	3.1
Western Macedonia	99	2.2
Thessaly	238	5.2
Central Greece	104	2.3
Western Greece	219	4.8
Crete	262	5.7
Ionian islands	51	1.1
North Aegean islands	92	2
South Aegean islands	87	1.9

Age standardization was performed using the 2011 Census as the reference population’s data to check a-posteriori that the sampled population was representative of the Greek population, as per the aim of the study (calculations are provided in the excel file provided in the Additional files 1 and 2). The population was stratified by 10 years and statistical analyses were performed. The sampled population was representative for the age groups 0–19, 20–65 and 65+, and, hence were used in the analysis. Furthermore, the prevalence of chronic diseases (surveyed) of the actual Greek population (as per census), through direct standardization, was compared to the prevalence found in the study population. The crude and adjusted odds ratios (OR) calculated by age group in total and by gender did not significantly differ, hence allowing increasing generalizability of the results.

### Inclusion – Exclusion criteria

Total HNNHS sample population included volunteers ≥6 months old that reside in Greece. Exclusion criteria included individuals (i), that did not speak Greek, (ii) women who were at that time breastfeeding or pregnant, (iii) members of the armed forces (including those that are currently undergoing their compulsory military service), (iv) individuals that reside in institutions (e.g. nursing homes, rehabilitation centers, hospice centers, psychiatric institutions, prisons, monasteries), (v) those that were unable to provide informed consent due to any cause (e.g., mental impairment, psychiatric condition, drug abuse, vision or hearing loss) unless a first degree relative was able to assist in the process.

### Data collection

Information was collected via a series of previously validated questionnaires, from the entire population sampled (details given in “Questionnaires in brief” section). All of the questionnaire types used in HNNHS are provided in supplementary tables, along with their validation references in Additional file 1: Tables S1-S3. Additional references are listed for those questionnaires that are not relevant in this study’s results.

Clinical examinations were performed on a subsample. More specifically, an initial interview took place at the volunteer’s house, with the use of a specially designed computer software (i.e. Computer Assisted Personal Interview (CAPI)), to minimize response biases and misclassification (minimize volunteer burden and maximize reliability of collected data). The list of questionnaires applied can be seen in the Additional file 1: Tables S1 and S2. In summary, the interviewing process included data on (i) demographics, (ii) quality of life (QoL), (iii) medical history (i.e. chronic & autoimmune diseases, depression, anxiety), (iv) breastfeeding, (v) vitamin and subscribed drug intake, (vi) memory impairment, (vii) eating habits, (viii) alcohol intake, (ix) smoking habits, (x) physical activity, (xi) sleeping habits, (xii) overall patient health, and (xiii) effects of economic crisis. The questionnaires were chosen according to the volunteer’s age, as designated by the study’s protocol (Additional file 1: Tables S1-S3).

A detailed 24-h dietary recall was obtained during this process. The volunteers were also interviewed for a second 24-h dietary recall via telephone 8–20 days after the first interview, selecting a different day, and non-consecutive, as specified by HNNHS study-protocol. Specific questionnaire structure and validated food atlases for food quantification were used depending on volunteer’s age (≥1.5–4 years old, ≥4- < 10 years old, ≥10- < 12 years old and ≥ 12 years old) in order to maximize response accuracy. More specifically, dietary intake data were collected using two automated multiple-pass 24-h dietary recalls and a Food Propensity Questionnaire (FPQ). To harmonize data collection, we based our food classification and description system on FOOdEx2 developed by EFSA [8], based on volunteers age (< 2 years old and ≥ 2 years old). Main differences between the two versions was the food list, (was shorter for the < 2 year old’s), as well as the frequency response section. The latter referred to the frequency of food intake over the last 30 days for volunteers < 2 years old, or to the past year for those ≥2 years old. Both FPQs were developed based on the Hellenic, European and International guidelines. Overall, the methods of dietary assessment were chosen as per EFSA recommendations for the harmonization of data across countries member states of the European Union [8]. Data on eating patterns and behaviors were also collected (timing of food intake, number of meals, activities performed during food consumption, place of consumption, and others) to account for their effects on individuals weight status as studies support [9–11]. The Nutrition Data System for Research (NDSR) (developed by the University of Minnesota) was used for nutrient analysis.

At the end of the interview, volunteers were provided with a list of questionnaires (hard copy) with specific instructions, to self-complete, based on the volunteer’s age and their primary response to disease state during the interviewing process (Additional file 1: Table S2). These were to be fulfilled within a specific time period, to further reduce volunteer burden (time related) and to decrease interviewer and response bias because of the nature of the nature of the questionnaires (sensitive personal information). These questionnaires included (i) qualitative FPQ (asked to be completed by all volunteers, as explained above), (ii) perceived stress scale, (iii) perception of health control, (iii) eating behavior (iv) chronic disease specific information (onset, treatment, medical follow ups, and others), (v) pregnancy and infantile information (i.e., smoking during pregnancy, number of children, weight gain per pregnancy, infant’s birth weight/length, breastfeeding (type & duration), and others), (vi) environmental exposure, (vii) social readjustment factors due to the economic crisis, (viii) asthma related information, and (vi) gastrointestinal disorders (the Greek version of Rome III FGID questionnaires for both children and adults was completed).

Interview based questionnaires and those to be self-completed were addressed to volunteers ≥12 years old. Questionnaires related to volunteers, less than 12 years old, were addressed to his/her parent or primary guardian.

In the case of volunteers being unable to self-respond (i.e., with inhibiting health complications, adolescents with lack of knowledge in specific questions) a parent/guardian was asked to assist in the interview. The economic crisis questionnaire was answered only by one adult member per household. Information on primary respondent, or on potential help received during the process was recorded (“interviewee assistant”). A small list of questionnaires where exempt from this procedure (where the main respondent has to be the volunteer himself), due to the nature of the related questions. These included questions on (i) memory impairment, (ii) screen time and alcohol use, (≥12 years - < 18 years), (iv) smoking habits (≥12 years - < 18 years) and (v) patient health questionnaire.

Completed questionnaires were handed to the participants nearest mobile unit or were given to the experienced field investigator (who performed their initial interview), when completed. To achieve a maximum response rate, the study’s trained personnel performed kind reminders via phone calls. A total of 3180 volunteers (2682 adults and 498 children and adolescents) completed all questionnaires (67% in total; 71% for adults and 62.6% for children & adolescents). Field investigators completed a quality control check-list upon checking the completed questionnaires.

Blood samples were taken from a sub-sample of the population. More specifically, all participants were invited to provide blood samples for biochemical – hematological evaluation. Of them, 1197 (26.2% of total population; 28.7% of adult population) agreed; no age distribution differences were found between the total population and those who provided blood sample (*p* = 0.677). Each of these individuals visited one of the 5 mobile units where medical and anthropometrics were completed (please see Additional file 1: Table S3). All samples were collected in the morning, between 8:00 and 10:00 am, upon having fasted for at least 10 h. To assure compliance all individuals were asked if they had fasted and when their last meal was.

Experienced field investigators were from various scientific fields (dietitians, physicians, sociologists as well as dietetic and medical students), and received specialized training on the HNNHS fieldwork protocol. These specialists were involved in the development, methodology and application of study questionnaires and protocol procedure attainment was assessed with quality control testing, during field-investigation.

### Ethical approval and consent form

The study was approved by the Ethics Committee of the Department of Food Science and Human Nutrition of the Agricultural University of Athens. It was also approved by Hellenic Data Protection Authority (HDPA). All members of the staff signed confidentiality agreements. Adult volunteers were asked to sign the consent form. For minors < 13 years of age the parent or primary guardian signed the form and for volunteers between 13 and 18 years of age the consent form was asked to be signed by both (parent/ guardian and volunteer).

### Questionnaires in brief

All questionnaires used in HNNHS, were derived based on a priori knowledge and from components of previously validated questionnaires. For this process. The outcome of interest and previous work performed in the Greek population were also considered.

For demographic characteristics (marital status, education, health insurance, employment, income and changes in employment and/or income during the economic crisis) components from NHANES [12], Behavioral Risk Factor Surveillance System (BRFSS) study [13] and NDNS [14], questionnaires were used.

The Quality of Life (QoL) questionnaire included components of (i) QoL and chronic pain components of the Healthy Days Module developed by the Center for Disease Control (CDC) [15], (ii) questions with regards to self-reported height, weight and oral health, from the Health Survey for England and the Activity Limitations Module (also CDC developed) [16].

Two questionnaires were developed for alcohol consumption; one for minors and the second for adults. For minors (≥12 years old and < 18 years old) the questionnaire was developed based on questions from the Youth Risk Behavior Survey [17], the European School Survey Project and other Drugs [18] and the Global School-based Student Health Survey (GSHS) [19]. For the adult questionnaire data from NHANES study [12], BRFSS [13], Arkansas Cardiovascular Health Examination Survey (ARCHES) [20] and Recommended Alcohol Questions by the National Institute on Alcohol Abuse and Alcoholism (NIAAA) [21] were used. Volunteers were classified as alcohol or non-alcohol consumers, based on their intake over the past 30 days. Frequency of alcohol intake among “consumers” was categorized as daily, weekly or monthly, based on their response on (i) total drinks per month consumed, (ii) drinks per week and/or (iii) drinks per month. For minors, the total number of individuals that reported having consumed an alcoholic drink at some point in life (and not just few sips) was reported.

As in the case of alcohol consumption, smoking habits questionnaire(s) were also based on volunteer’s age. In particular, for adults questionnaires used were from the NHANES [12] and BRFSS [13] studies; for minors from the Youth Behavior Survey [17], NHANES [12] and the European School Survey Project on Alcohol and other Drugs [18]. Volunteers were grouped into (i) current smokers, if they responded that they had smoked the past month, (ii) ever smokers, if they had smoked at any point in their life, and (iii) non-smokers, if they had never smoked. Frequency of smoking, among current smokers, was also recorded as “daily” or “sometimes”. Among minors, the question referred as to whether they had ever tried to smoke (aged up to 19 years).

Weight status was evaluated according to BMI. Body mass Index (BMI) is defined as the weight (in kg) over height (in meters squared). Cut-offs used (all in kg/m^2^) for assessment are widely used and are the following (adults): < 18.5, underweight; 18.5–24.9, normal weight; 25–29.9, overweight; > 30, obese.

Physical activity has a well-known role as a health determinant hence the aim was to assess physical activity levels in all ages. Questionnaires on physical activity were modified based on age groups as per a priori knowledge, including (i) ≥2- < 12 years old of the questionnaire was based on questions from the NHANES survey [22] and Preschool-aged Children Physical Activity Questionnaire (Pre-PAQ Home Version) [23] (ii) ≥12- < 18 years old, the International Physical Questionnaire – Adolescents (IPAQ-A) [24], (iii) ≥18 years- < 65 years old the IPAQ short form was used [25] and (iv) for ≥65 years of age a modified version of the IPAQ has been suggested [26]. Preliminary results reported in this study include level of physical activity as perceived by the adult volunteers (sedentary, low, moderate and active) or by the primary care giver if the volunteer was < 12 years old.

Information about medical history for disease prevalence among the Greek population, related medical treatment and insurance coverage were collected. The synthesis of this questionnaire was based on the National Health Survey, NHANES [12], ARCHES [20], and the Million Women Study [27]. The definition of clinical investigated outcomes was based on the International Classification of Diseases (ICD)-10th version, recorded by experienced study investigators. Diabetes was defined as fasting blood glucose> 125 mg/dl or if on diabetic medication; dyslipidemia if total triglycerides> 150 mg/dl and/or total cholesterol> 200 mg/dl or on lipid-lowering medication; hypertension as average blood pressure greater or equal to 140/90 mmHg, or on antihypertensive treatment.

Further details on specific disease states (hypertension, dyslipidemia, diabetes) with specific questionnaires [20], were collected once the volunteer declared as having such a condition. In particular, data on prevalence of Chronic Obstructive Pulmonary Disease (COPD) was obtained using the COPD Population Screener [28] and Asthma using the questionnaire from the Hellenic Thoracic Society (for adults), and the Greek version of the questionnaire International Study of Asthma and Allergies in Childhood (ISAAC) [29] (minors 6–18 years old). Following a literature review the Rose Questionnaire for Angina [30] and the Edinburgh Claudication Questionnaire were used in HNNHS [31].

Additional the types of questionnaires used in the study can be viewed in the Additional files 1 and 2 and they included information on breastfeeding, drug and supplement use, memory impairment (≥45 years old), eating habits and behavior (as previously reiterated), sleeping habits, data on depression, stress (acute and chronic) & health locus of control, gestational & child- birth related questions, environmental exposure, functional gastrointestinal disorders, vitamin D intake status & sun exposure, and economic crisis, to acquire adequate and substantial information on the population’s exposures and risks. Details for each of these questionnaires will be provided upon analysis.

### Clinical/ physical evaluation and biochemical variables

HNNHS also included physical examination (temperature, spirometry, blood pressure, etc.), anthropometry (weight, height, waist and hip circumference, body composition, and grip strength), and several blood tests (glucose, HbA1c (diabetics), insulin, total lipid profile, thyroid hormones, thyroglobulin, PTH, complete blood count, folic acid, iron, ferritin, B_12_, 25OH-vitamin D, creatine, urea, albumin, total protein, ALT, AST, bilirubin, uric acid, calcium, magnesium, manganese, selenium, hs-CRP, cortisol, and heavy metals, namely As, Cd, Co, Hg, Mo, Pb, Pt, Sb, W, Zn, Ce, La, Th, U) in a subsample of the population, to examine correlations with various health indices in later analyses (Additional file 1: Table S3).

### Statistics

Prior to analysis, data were cross checked for missing values and outliers. Missing information was corrected if the information was derived from other questionnaires and/or measurements (non-reported values of weight and height were completed if the individual was measured at the CAPI). Also, individuals responding as non-diseased but reported taking a disease related medication, were classified as with disease outcome. Baseline socio-demographic are presented as frequencies and percentage (N, %) per gender. Variables of interest are presented in total and per gender and age-group (i.e., population’s weight status, smoking, alcohol, physical activity, prevalence of chronic disease), while physical activity is presented by specific age groups (as per questionnaires). Chi-square test was used to assess gender differences by age group for weight status, smoking and alcohol intake, and for total prevalence of chronic disease by gender. Tukey’s paired means test was used to detect differences between age groups (for each chronic diseases). All reported *p*-values were based on two-sided hypothesis tests, with significance level at 5%. The statistical models were computed using STATA 12.0 (STATA corp. Texas).

## Results

### Demographic data

The sample was distributed in all different regions of Greece (Table 1). 47.2% was in the region of Attica, 18.5% Central Macedonia, and the rest of the sample being scattered through various regions of Greece (1.3% Epirus, 4.2% Eastern Macedonia and Thrace, 3.1% Peloponnese, 2.2% Western Macedonia, 5.2% Thessaly, 2.3% Central Greece, 4.8% Western Greece, 5.7% Crete, 1.1% Ionian islands, 2% North Aegean and 1.9% South Aegean).

The total number of participants is 4574 volunteers of which 1943 were males and 2629 females. Table 2 shows distribution per gender, age and socioeconomic parameters. Age distribution was representative of the 2011 Census, with 19% (*N* = 869) of the sampled population being 0–19 years old, 67% (*N* = 3064), 20–64 years old, and 14% (*N* = 639) were ≥ 65 years old. Marital status was as follows: 40.6% of the population was unmarried (43.3% males and 38.5% females), 48.4% married (51.4% males and 46.4% females) and 0.1% having a cohabitation agreement 6.2% were widowers (2.2% males and 9.2% females), 3.8% divorced and 0.7% separated.

**Table 2:** Volunteer baseline socio-demographic characteristics by gender

	Males		Females	
	N	%	N	%
	1943	42.5	2629	57.5
Age				
0–19	426	21.9	443	16.9
20–64^a^	1259	64.8	1805	68.7
*20–39*	*797*	*41.0*	*1040*	*39.6*
*40–65*	*462*	*23.8*	*765*	*29.1*
65+	258	13.3	381	14.5
Marital status				
Unmarried	841	43.3	1012	38.5
Married	998	51.4	1217	46.3
Cohabitation agreement	2	0.1	2	0.1
Widower	43	2.2	241	9.2
Divorced	47	2.4	127	4.8
Separated	10	0.5	23	0.9
Don’t know	–	–	1	0
Refused	–	–	4	0.2
Educational level				
No or little education	25	1.6	90	4
Primary school	128	8.2	224	9.9
Gymnasium	81	5.2	99	4.4
Lyceum	418	26.7	621	27.3
Technical school	133	8.5	57	2.5
Private college (Post Lyceum)	114	7.3	204	9
University degree (AEI)	336	21.5	517	22.7
University degree (TEI)	144	9.2	219	9.6
Master’s degree	109	7	188	8.3
PhD	31	2	22	1
Refused	4	0.3	3	0.1
Net monthly income (€)				
≤ 300	76	3.9	106	4
301–650	148	7.6	285	10.8
651–850	171	8.8	264	10
851–1050	237	12.2	283	10.8
1051–1250	172	8.9	236	9
1251–1500	178	9.2	237	9
1501–1900	222	11.4	264	10
1901–2400	183	9.4	231	8.8
2401–3800	177	9.1	202	7.7
> 3801	51	2.6	59	2.2
Don’t know	122	6.3	214	8.1
Refused	204	10.5	246	9.4
Health insurance				
Uninsured	156	8	162	6.2
Insured, private	91	4.7	105	4
Insured, public	1511	77.8	2071	78.8
Insured, both private and public	157	8.1	252	9.6
Don’t know	10	0.5	20	0.8
Refused	4	0.2	3	0.1

^a^The sampled population (N%) in the age group 20–64, was further categorized to 20–39 years and 40–65 to cross-reference with further analysis performed in these sub-categories

Educational level greatly varied with approximately 32% having a University degree or greater, 7.1% had completed secondary education. Approximately 17% of the population had limited to low education, 27.1% completed lyceum (12 years of schooling), 5% technical secondary school and 8.3% private post-lyceum college. A large percentage of the population (78.3%; 77.8% males and 78.8% females) reported having public health insurance whereas only 4.3% had private insurance and 8.9% both types. A total of 8% males and 6.2% females were not insured (Table 2).

In terms of net monthly income (Table 2), 13,5% had low income (<€300–850), 11.4% had €851–1050, approximately 18% had moderate high income (€1051–1500), 10.6% had €1501–1900, 9.1% had €1901–2400, and 10.7% had high income (€2401–3800 and > €3801).

### Weight status and behavioral data

Sample’s self-reported weight status in total by age group (> 20 years old) and gender based on Body Mass Index (BMI) can be found in Table 3. A total prevalence of 47.5% of the adult population was overweight (32%) and obese (15.5%), with the prevalence increasing with age in both genders. A significant body weight status difference was found in each age group, with males having a higher prevalence of overweight compared to females (*p* < 0.001) in all age groups.

**Table 3:** Population’s weight status in total by age group and gender based on Body Mass Index (BMI) categorization

Weight status categorization^d^	Total		By age group^e^ and gender					
	Total adult population^e^		20-39^a^		40-64^b^		65+^c^	
			N (%)		N (%)		N (%)	
	N	%	M	F	M	F	M	F
Underweight	175	4.7	12 (1.5)	88 (8.5)	5 (1.1)	25 (3.3)	8 (3.1)	37 (9.8)
Normal weight	1772	47.9	420 (52.7)	722 (69.5)	139(30.2)	335 (43.8)	60 (23.3)	94 (24.8)
Overweight	1183	32.0	285 (35.8)	160(15.4)	212 (46.0)	244 (31.9)	127 (49.2)	154 (40.6)
Obese total	572	15.5	80 (10.0)	69 (6.6)	105 (22.8)	161 (21.1)	63 (24.4)	94 (24.8)

*N (%)* Frequency (percentage), *M* males, *F* Females

By gender: % of males or females in question compared to total number of males or females, respectively

^a^Chi square test for difference in weight status between genders in 20–39-year-old group (*p* < 0.001)

^b^Chi square test for difference in weight status between genders in 40–65-year-old group (*p* < 0.001)

^c^Chi square test for difference in weight status between genders in 65+ year-old group (*p* < 0.006)

^d^Based on BMI (kg/m^2^) categorization: < 18.5 = underweight; 20–25 = normal weight; > 25–30 = overweight; > 30 = obese

^e^Study population ≥ 20 years of age; Chi square test for difference in weight status between age groups in total (*p* < .001) and per gender (*p* < .001)

Frequency of alcohol consumption among adults was 72.4% (Table 4), with approximately 7% reporting daily consumption, 33% weekly and 60% on a monthly basis. A significant greater percentage of males reported of being alcohol consumers than females (81.1% compared to 67%, respectively; *p* < 0.001) and being more frequent alcohol consumers as well (*p* < 0.001). Among minors (12 to 19 years of age, inclusive), 111 out of 340 individuals (32.6%) reported as having consumed an alcoholic drink at some point before, and not only a few sips (Table 4). No significant differences were found between genders among minors in alcohol consumption (*p* = 0.121).

**Table 4:** Frequency of alcohol consumption habits in minors and adults in total and by gender

Adults (20+ years)	Alcohol consumption*						
	Total		Males		Females		Level of significance ^a^
	N	%	N	%	N	%	
The past 30 days*							
No	998	26.9	285	18.8	713	32.8	*p* < 0.001
Yes	2685	72.4	1229	81.1	1454	67.0	
Frequency							
Everyday	183	6.8	128	10.4	55	3.8	*p* < 0.001
Weekly	874	32.6	456	37.1	418	28.8	
Monthly	1628	60.6	645	52.5	981	67.5	
Minors (12–19 years) **							
Ever consumed							
No	229	67.4	142	89.3	153	84.5	*p* = 0.121
Yes	111	32.6	17	10.7	28	15.5	
Don’t know	1	0.5					
Refused	–	–					

*For adults (20 + years of age: *N* = 3705 in total, 7 missing): any alcohol consumption the past 30 days and frequency of consumption

**For minors (12–20 years of age, *N* = 340, 159 males and 181 females): whole alcoholic drink consumed at some point in life (and not just few sips). 66 minors were < 18 years and 45 18 &19 years old

^a^Tested via chi square test for gender differences in adult population (20 years +) and in minors (up to 19 years); *p < 0.05; **p < 0.01; ***p < 0.001;

Smoking frequency in the total population among adults and minors, per gender, is being shown in Table 5. Approximately 34% of the population were current smokers, whereas 50.9% reported on having smoked at some point in their life. Significant gender differences were found in both cases with a higher proportion of males reporting to have smoked (59% compared to 44%) or of being current smokers (38.3% compared to 30.8; *p* < 0.001 for all). Among current smokers 87.3% reported to smoke daily with a borderline difference found between genders (*p* = 0.046). A total of 22% of minors (up to 19 years of age, inclusive) reported of having tried to smoke at some point. No significant gender differences were found (*p* = 0.229).

**Table 5:** Frequency of smoking habits in total population among adults and minors by gender

Adults (20+ years)	Smoking**						
	Total		Males		Females		Level of significance^b^
	N	%	N	%	N	%	
Ever smoked							
No	1935	52.4	620	41.0	1215	56.0	*p* < 0.001
Yes	1878	50.9	893	59.0	955	44.0	
The past 30 days*							
No	2433	65.7	934	61.5	1497	68.5	*p* < 0.001
Yes	1252	33.8	580	38.2	672	30.8	
Frequency							
Every day^a^	1093	87.3	519	89.5	574	85.4	*P* = 0.046
Some days^a^	158	12.6	60	10.3	98	14.6	
Don’t know	1	0.1					
Refused	–	–					
Minors (10–19 years)							
Ever smoked							
No	100	76.3	33	70.2	67	79.8	*p* = 0.229
Yes	29	22.1	12	25.5	17	20.2	
Don’t know	1	0.8					
Refused	1	0.8					

*For adults (> 19 years of age: 3705 in total): ever smoking; for minors specified if they even tried it (then response yes)

^a^For adults smoking the past 30 days (frequency (%) of smoking for smokers *N* = 1252)

^b^Tested via chi square test for gender differences in adult population (20 years +) and in minors (up to 19 years); *p < 0.05; **p < 0.01; ***p < 0.001;

Preliminary results of physical activity level were self-reported as sedentary, low activity, moderately and very active (Table 6). The highest proportion of the population being very active was in young children (2–12 years old, 68.6%) and among adolescents (48.5%). Twenty – 5 % (25%) of adults aged 18–65 and > 65 years old reported being very active whereas 20% of the elderly (> 65) reported of having a completely sedentary lifestyle.

**Table 6:** Physical activity levels among different age groups based on self-reported data

Physical activity*	≥2 - < 12 years		≥12 - < 18 years		≥18 - < 65 years		≥65 years old	
	N	%	N	%	N	%	N	%
Sedentary way of life	–	–	–	–	–	–	128	20
Low activity	15	3.2	24	11.7	584	18.3	117	18.3
Moderate active, average	126	26.7	74	35.9	1357	42.4	205	32.1
Very active	324	68.6	100	48.5	812	25.4	160	25
Don’t know	–	–	1	0.5	2	0.1	1	0.2
Refuse to respond	–	–	–	–	2	0.1	–	–

*Individuals were asked to report their perceived physical activity status or to state their child’s if they responded on their behalf

### Prevalence of chronic disease

In Table 7, the prevalence of various chronic diseases is presented in total and per age group (20–39, 40–64, and 65+) in adults. In each category, gender specific rates can also be viewed. The highest prevalence (16.7%) was reported for hyperlipidemia (increased cholesterol or triglycerides), with prevalence increasing in both genders with age (Tukey’s test *p* < 0.001 between groups). The same pattern was found for hypertension with the prevalence mounting to 56% (51.2% in males, 61% in females; *p* < 0.05) in the elderly compared to 1.7% in adults aged 20–39 and 17.3% in the 40–65 age group (Tukey’s test not significant). Accordingly, age patterns were seen in all CVD (CHD, angina, MI, heart failure, arrhythmia and stroke), with significant age group differences found only in heart failure (Tukey’s test *p* = 0.014 for 65+ compared to 20–39 years). Diabetes prevalence and osteoporosis was also considerably higher in the older age group (16,8%) compared to 3.8% in total population and 16.2% compared to 5.4%, respectively. Only osteoporosis was significantly different between age groups (*p* < 0.001 for 65+ and 20–39 and 40–64). The prevalence of thyroid disease was high in all age groups, especially in females and significantly different between the 65+ and 20–39-year-old age groups (Tukey’s test *p* = 0.026). A significant difference was also found in cancer prevalence between the older and younger adult age groups (Tukey’s test, *p* = 0.033).

**Table 7:** Prevalence of chronic disease in adult population sampled, in total, by gender and by gender and age group

Presence of disease/condition	Total				By gender and age group^a^					
	Total sample		By Gender^b^ N (%)		20–39 N (%)		40–64 N (%)		65+ N (%)	
	N	%	M	F	M	F	M	F	M	F
Increased cholesterol or triglycerides^c^	765	16.7	297*** (15.2)	468 (17.8)	62** (7.8)	48 (4.6)	127 (27.6)	226 (29.5)	103* (39.9)	183 (48.3)
Don’t know	175	3.8								
Hypertension	608	13.3	241 (12.4)	367 (14.0)	21* (2.6)	11 (1.1)	88 (19.1)	124 (16.2)	132* (51.2)	231 (61.0)
	47	1.0								
Coronary Heart Disease	69	1.8	53*** (3.4)	16 (0.7)	0 (0)	1 (0.1)	17 (3.7)	1 (0.1)	36*** (14.0)	14 (3.7)
Don’t know	32	0.8								
Angina	36	0.9	19 (1.2)	17 (0.8)	6 (0.8)	4 (0.4)	6 (1.3)	2 (0.3)	7 (2.7)	10 (2.6)
Don’t know	31	0.8								
Myocardial Infarction (Heart attack)	49	1.3	37 (2.4)	12 (0.5)	0	0	16** (3.3)	5 (0.7)	21*** (8.1)	7 (1.9)
Don’t know	13	0.3								
Heart failure	42	1.1	16 (1.0)	26 (1.1)	0	3 (0.3)	2 (0.4)	8 (1.1)	14 (5.0)	15 (4.0)
Don’t know	27	0.7								
Arrhythmia	295	7.7	91** (5.8)	204 (9.0)	21 (2.6)	48 (4.6)	25 (5.4)	71 (9.3)	45 (17.4)	78 (20.6)
Don’t know	42	1.1								
Stroke	41	1.1	18 (1.1)	23 (1.0)	1 (0.1)	2 (0.2)	3 (0.7)	4 (0.5)	14 (5.4)	17 (4.5)
Don’t know	11	0.3								
Cancer	53	1.2	14** (0.7)	39 (1.5)	3 (0.4)	1 (0.1)	3** (0.6)	28 (3.7)	8 (3.1)	10 (2.6)
Don’t know	8	0.2								
Diabetes (Type I & II)^d^	162	3.6	73 (3.8)	89 (3.4)	3 (0.4)	4 (0.4)	27** (5.9)	21 (2.7)	42 (16.3)	64 (16.9)
Don’t know	24	0.5								
Thyroid (any type of condition)^e^	629	13.8	93*** (4.8)	536 (20.4)	36*** (4.5)	160 (15.4)	26*** (5.6)	248 (32.4)	24*** (9.3)	113 (29.8)
Don’t know	102	2.2								
Asthma	184	4.0	69 (3.6)	115 (4.4)	40 (5.0)	48 (4.6)	8* (1.7)	37 (4.8)	6 (2.3)	20 (5.3)
Don’t know	16	0.4								
Chronic Obstructive Pulmonary Disease (COPD)	63	1.6	25 (1.6)	38 (1.7)	5 (0.6)	8 (0.8)	9 (2.0)	15 (2.0)	11 (4.3)	15 (4.0)
Don’t know	77	0.6								
Chronic kidney disease	27	0.6	13 (0.7)	14 (0.5)	3 (0.4)	1 (0.1)	2 (0.4)	4 (0.5)	8 (3.1)	9 (2.4)
Don’t know	3	0.1								
Osteoporosis^f^	206	5.4	13*** (0.8)	193 (8.3)	1 (0.1)	3 (0.3)	4*** (0.9)	95 (12.3)	8*** (3.1)	95 (25.8)
Don’t know	79	2.1								
Arthritis/ Rheumatoid disease	324	7.1	65*** (3.3)	259 (9.9)	9* (1.1)	23 (2.2)	28*** (6.1)	106 (13.9)	28*** (10.8)	128 (33.8)
Don’t know	83	1.8								
Crohn’s disease or Ulcerative colitis	16	0.4	6 (0.3)	10 (0.4)	2 (0.2)	3 (0.3)	1 (0.2)	6 (0.8)	3 (1.2)	1 (0.3)
Don’t know	6	0.1								
Irritable Bowel Syndrome (IBS)	317	6.9	53*** (2.7)	264 (10.1)	25*** (3.1)	105 (10.1)	18*** (3.9)	121 (15.8)	9* (3.5)	35 (9.2)
Don’t know	46	1.0								
Depression	180	4.2	42*** (2.3)	138 (5.6)	15 (1.9)	33 (3.2)	12*** (2.6)	62 (8.1)	15 (5.8)	42 (11.1)
Don’t know	63	1.5								
Chronic Stress	495	11.6	128*** (7.1)	367 (14.9)	56*** (7.0)	143 (13.8)	42*** (9.1)	134 (17.5)	25** (9.7)	78 (20.6)
Don’t know	39	0.9								

By gender: % of males or females who reported as having the outcome in question compared to total number of males or females, respectively

By age-group: Number of outcomes reported per gender in each age-group (%)

^a^Tested via chi square test for gender differences by age group

^b^tested via chi square test for gender differences in total sample; *p < 0.05; **p < 0.01; ***p < 0.001

^c^3.5% of the sample replied that they do not know for cholesterol; < 1% for hypertension, coronary heart disease, angina, myocardial infarction (0.3), stroke (0.3), heart failure,; arrhythmia (1.1%), diabetes (0.53), 2.2% for any thyroid disease, asthma (0.35%), chronic obstructive pulmonary disorder (0.63%). Kidney failure (0.1%), 2.1% for osteoporosis, 1.8% for arthritis, 0.1% for Crohn’s disease, 1.0% for irritable bowel syndrome, 1.5% for depression, 0.91% for chronic stress

^d^Prevalence for type I diabetes: 3/4754

^e^0–19 age group: for thyroid disease: Males (1.6%) and females (3.4%); For asthma: Males (3.5%), Females (2.3%); For chronic stress: Males 1.55%, females 4.3%

^f^Out of which 13 osteopenia

### Gender differences and chronic disease

Significant gender differences were found in hyperlipidemia, arrhythmia, cancer, thyroid disease, osteoporosis, arthritis/rheumatoid arthritis, irritable bowel syndrome, depression, and chronic stress, with females having a significantly higher proportion in each one of them. Prevalence of asthma and cancer was also higher in females, more specifically in the 40–64 age group (4.8% vs. 1.7%; *p* < 0.05 and 3.7% vs. 0.6%; *p* < 0.01, respectively). Gender difference was also found in CHD with males having a higher prevalence in the total adult sample and in the older group (*p* < 0.001, for all). The prevalence of MI did not differ in the total sample but was significantly higher in males over 65 years old than females in the same age group (9.1% vs. 1.9%; *p* < 0.001). Diabetes mellitus was significantly higher in males aged 40–64 years old than females of the same age group (5.9% vs. 2.7%; *p* < 0.01).

## Discussion

The HNNHS was set up in 2013 with the aim to provide comprehensive, nutrition and health information, on a representative sample of the Greek population. Preliminary results of the HNNHS study showed an elevated prevalence of overweight and obesity in adults as well as dyslipidemia and hypertension. Among the adult population prevalence of overweight & obesity was almost 47%, significantly varying by gender, 17% of the total population had dyslipidemia, 13% hypertension and about 4% had diabetes and 14% were affected by a form of thyroidism. All outcomes significantly increased with age with prevalence of dyslipidemia and hypertension reaching 45 and 57% in the elderly, respectively. Furthermore, the prevalence of osteoporosis in Greek women over 65 year of age was 25.8%, a disease that is highly preventable.

In more detail, prevalence of overweight and obesity as well as chronic diseases increased with age with males having overall a higher weight status than females. This is in accordance with data from NHANES showing increased levels of obesity in adults, by sex, age and ethnicity. Hyperlipidemia prevalence in 2011 in Greece was 15% [32], and results from the ATTICA study reported that 1 in 2 adults (45 ± 15) years old was dyslipidemic [33].This is in accordance with current results from HNNHS (44,8% in total; 39.9% in males and 48.3% in females). High levels of hypertension and hyperlipidemia were also found in other studies [34, 35]and policies targeting the reduction of these public health outcomes are warranted as were developed by other countries upon findings [34, 35]. Participation rate was higher in females than males, as has been reported in most European countries [36].

The proportion of alcohol consumption and current smoking status was high, although the latter, prevalence of smoking, was lower compared to previous findings in the Hellenic population [37]. An alarming proportion of minors had tried alcohol or had smoked at some point. Smoking is a known risk factor for many chronic diseases, including cardiovascular disease, many forms of cancer, asthma and COPD. Alcohol, although has been found to have protective effects on CVD, when consumed in moderation [38], it is forbidden in minors.

Regarding arterial hypertension, the present study’s preliminary results are comparable with other studies where hypertension was self-reported (13.1% vs. 13.3%, respectively, *n* = 5003) [39]. As hypertension is a common risk factor of cardiovascular disease, data on level of awareness is warranted. Efstratopoulos et al., found an awareness level of 60.2% among Greek hypertensive individuals [40], therefore, further investigation is warranted. The prevalence of hypertension in the NHANES study, for those ≥20 years old was also close to the EPIC and HYPERTENSHELL studies (33.5%) [41]. A 4% prevalence of diabetes mellitus was found in this study, reaching 6.3% for adults over 30 years of age, compared to 7–11% prevalence reported in Greece among adults [33, 42, 43]. HNNHS included information on thyroid and renal function, for which there are no data available in the Greek population. Respective prevalence levels of 13.7 and 0.6% of those ≥20 years old were reported. The increased prevalence in all types of thyroid conditions (hypothyroidism, hyperthyroidism, Hashimoto thyroiditis), especially among women, underlies the value of HNNHS and stresses the need to further investigate risk factors linked to this outcome, such as iodine and vitamin D status, as well as nutritional intake and search for deficiencies. The prevalence of cardiovascular disease, the leading cause of mortality worldwide, found in the study population was 13.9%, in total. This included 7.7% of the total sample that reported having arrhythmia, 1.8% coronary heart disease, 1.3% myocardial infarction, 0.9% angina, 1.1 heart failure, and 1.1% had suffered a stroke.

Furthermore, an increased level of stress-associated disorders including chronic perceived stress (11.6%), depression (4.2%), Crohn’s disease (0.4%) or ulcerative colitis (0,4%), and irritable bowel syndrome (6.9%) were found. These outcomes may be associated with the economic crisis seen in Greece over the past years but can also be linked to various nutritional and behavioral factors, that need to be examined. Interestingly, data with regards to perceived change in household budget show that most volunteers perceived change being more severe in 2012 (23.2%) than 2011 (18.3%) and 2013 (12.6%). Details that may have affected these stress-associated disorders, remain to be investigated.

### Limitations

Due to the cross-sectional nature of the study, no causal relationships can be formulated. Also, the data presented and analyzed in this first report are from reported data. However, experienced field investigators checked the data and recorded clinical outcomes based on ICD-10th version codes. Furthermore, sensitive personal questions, were self-completed to decrease reporting bias. All clinical outcome data were further cross-checked with other related questions, ie, medications, in order to accurately code the participants and decrease misclassification. Reporting of data in more depth and comparison with other past small, non-nationally representative surveys in Greece are beyond the scope of this first methodological publication and will be described elsewhere.

### Strengths

Health surveys as HNNHS can reveal target groups in need for prevention strategies according to educational level, employment and marital status, area of residence in a subnational level, and health behavior [40, 44]. HNNHS, is the first national representative study performed in Greece to assess nutrition and health status of the population including all age groups. Questionnaires used were constructed after performing an extensive literature review and based on other validated questionnaires that have been used in other large national studies and in the Greek population. Another strength is the synergistic action of multiple health care specialists in study design, filed work and data analysis. Furthermore, the use of the especially designed computer software, CAPI, increases reliability of collected data, since it reduces response bias, misclassification and volunteer burden. Measurements, clinical assessment and blood tests performed in a subsample of the population will be used to further validate the preliminary results presented here.

## Conclusions

The HNNHS study aims to evaluate the health of the Greek population. The data presented provide a preliminary overview of demographic and lifestyle data of the population. We envision that this study will provide valuable information regarding the health of the Greek population and that it will become a rolling program that will facilitate the development and evaluation of public health policies addressing key risk factors that impact on the health of the Greek population.

## Additional files

Additional file 1: **Table S1.** List of questionnaires applied to volunteers according to age during the initial interview. **Table S2.** List of questionnaires to be self-completed at home, according to age. **Table S3.** List of exams and questionnaires applied to volunteers, according to age, during their visit to the mobile unit. (DOCX 38 kb)

Additional file 2: 3 worksheets (All, Males, Females) for age adjusted chronic disease calculations, standardized by Hellenic population distribution (2011 Census). (XLSX 35 kb)

## Abbreviations

ARCHES: Arkansas Cardiovascular Health Examination Survey
BRFSS: Behavioral Risk Factor Surveillance System
CAPI: Computer Assisted Personal Interview
CDC: Centre for Disease Control
CHD: Coronary Heart Disease
COPD: Chronic Obstructive Pulmonary Disease
CVD: Cardiovascular disease
DM: Diabetes Mellitus
EFSA: European Food Safety Authority
EPIC: European Prospective Investigation into Cancer and Nutrition
FGID: Functional Gastrointestinal Disorders
FPQ: Food Propensity Questionnaire
GSHS: Global School-based Student Health Survey
HDPA: Hellenic Data Protection Authority
HNNHS: Hellenic National Nutrition and Health Survey
IPAQ: International Physical Activity Questionnaire
ISAAC: International Study of Asthma and Allergies in Childhood
MI: Myocardial Infarction
MRC: Medical Research Council
NatCen: NatCen Social Research
NDNS: National Diet and Nutrition Survey
NDSR: Nutrition Data System for Research
NHANES: National Health and Nutrition Examination Survey
NIAAA: National Institute on Alcohol Abuse and Alcoholism
OR: Odds ratio
QoL: Quality of Life
USA: United States of America
