# Supplementary material for: Aims, design and preliminary findings of the Hellenic National Nutrition and Health Survey (HNNHS)
Source: BMC Med Res Methodol. 2019 Feb 20;19:37. doi: 10.1186/s12874-018-0655-y (PMC6383253; doi:10.1186/s12874-018-0655-y)
Supplement: Supplementary file 1 — Table S1. List of questionnaires applied to volunteers according to age during the initial interview. Table S2. List of questionnaires to be self-completed at home, according to age. Table S3. List of exams and questionnaires applied to volunteers, according to age, during their visit to the mobile unit. (DOCX 38 kb) [file 12874_2018_655_MOESM1_ESM.docx]

**Table S1. List of questionnaires applied to volunteers according to age during the initial interview.**

| Questionnaire (by sequence of application to volunteers) | Application | References* |
| --- | --- | --- |
| Demographics | ≥6 months | 12, 13, 14 |
| Quality of life | ≥6 months | 15, 16 |
| Medical history | 1. ≥20 years 2. <20 years | 12, 20, 27 |
| Gynecological history | ≥8 years (women only) | 12, 27 |
| Breastfeeding history | ≥6 months - <6 years | 12 |
| Drug and supplement use | ≥6 months | 12, 20 |
| Memory Impairment Screen | ≥45 years | Buschke et al., 1999 |
| 24hr recall (1^st^) | ≥6 months | Blanton et al., 2006 |
| Eating habits | 1. ≥6 months - <12 years 2. ≥12 years | 12, 14, Bryant et al., 2008; Golan et al., 1998; Sorensen et al., 1998 |
| Alcohol consumption | 1. ≥12 years - <18 years 2. ≥18 years | 1. 17, 18, 19 2. 12, 13, 20, 21 |
| Smoking habits | 1. ≥12 years - <18 years 2. ≥18 years | 1. 12, 17, 18 2. 12, 13 |
| Physical Activity Questionnaire | 1. ≥2 years - <12 years 2. ≥12 years - <18 years 3. ≥18 years - <65 years 4. ≥65 years | 1. 22, 23 2. 24 3. 25 4. 26 |
| Sleeping habits | ≥6 months | 12, National Survey of children’s Health Questionnaire; Health Professionals Follow-Up Questionnaire |
| Patient Health Questionnaire | ≥18 years | Spitzer et al., 1999. |
| Economic crisis | ≥6 months | Economou et al., 2013. |

***Numbers designate references as stated in original text.**

**Table S2. List of questionnaires to be self-completed at home, according to age.**

| Questionnaire  (by sequence of application to volunteers) | Application | References* |
| --- | --- | --- |
| Food Propensity Questionnaire | ≥6 months - <2 years  ≥2 years | Willett et al., 2013; EFSA, PANCAKE for kids  Subar et al., 2006;  EFSA, PANCAKE for adolescence, adults-elderly. |
| Perceived Stress Scale | ≥18 years | Cohen et al., 1983  [(www.mindgarden.com/documents/PerceivedStressScale.pdf)](https://www.google.com/url?sa=t&rct=j&q=&esrc=s&source=web&cd=1&cad=rja&uact=8&ved=2ahUKEwio58KewYbgAhWMCuwKHbDxDfQQFjAAegQICRAC&url=http%3A%2F%2Fwww.mindgarden.com%2Fdocuments%2FPerceivedStressScale.pdf&usg=AOvVaw3rDQff2pc1shL-qsm8jhso) |
| Health Locus of control (Perception of health control?) | ≥18 years | Karademas et al., 2009 |
| Eating behavior | ≥1 year - <12 years  ≥12 years | Wardle et al. 2001); (Birch et al. 2001) |
| Dyslipidemia | <12 years  ≥12 years  (applied if volunteer has high cholesterol and/ or triglycerides) | 20 |
| Hypertension | <12 years  ≥12 years (applied if volunteer has hypertension) | 20 |
| Diabetes | <12 years  ≥12 years (applied if volunteer has diabetes) | 20 |
| Questionnaire with regards to the birth of children | ≥18 years - <65 years  (women with biological children) | Cancer Research UK and National Health Service; The Nurses' Health Study Questionnaire; <http://www.cdc.gov/nchs/nhanes.htm>]; http://www.ich.gr (πρόσβαση 04/2013). |
| Environmental exposure | ≥18 years | http://www.atsdr.cdc.gov/csem/exphistory/docs/exposure_history.pdf |
| Social Readjustment Rating Scale (SRRS) | ≥18 years | Holmes et al., 1967 |
| Asthma | ≥6 years - <12 years  ≥12 years - <18 years | 29  http://www.myasthma.gr (accessed 06/2013); (Papageorgiou et al. 1997); (Burney et al. 1994); The European Community Respiratory Health Survey. |
| Greek Version of Rome III Questionnaires for Functional Gastrointestinal Disorders in children and adolescents  Greek Version of Rome III Diagnostic Questionnaire for the Adult Functional GI Disorders | 1. ≥4 years - <10 years   ≥10 years - <18 years | <https://theromefoundation.org/products/copyright-and-licensing/>  (Drossman 2006) |

***Numbers designate references as stated in original text.**

**Table S3. List of exams and questionnaires applied to volunteers, according to age, during their visit to the mobile unit.**

| Procedure, Measurement, Test or Questionnaire (by sequence of application to volunteers) | Application |
| --- | --- |
| Salivetes collection | ≥18 years |
| Checking self-completed questionnaires completion | ≥6 months |
| Questionnaire regarding the preparation for the medical/ anthropometric evaluation tests | ≥6 months |
| Temperature | ≥6 months |
| Blood pressure | ≥3 years |
| Blood tests  (glucose, HbA1c (diabetics), insulin, total cholesterol, triglycerides, LDL, HDL, TSH, fT4, thyroglobulin, Anti-TSHR, PTH, complete blood count, folic acid, iron, ferritin, B12, creatine, urea, albumin, total protein, ALT, AST, bilirubin, uric acid, calcium, magnesium, hs-CRP, Vitamin D, cortisol (≥18 years), heavy metals (≥18 years) | ≥3 years |
| Length | <2 years |
| Height | ≥2 years |
| Head circumference | <5 years |
| Body weight | ≥6 months |
| Body Composition (Bioelectrical Impedance) | ≥3 years |
| Waist circumference | ≥3 years |
| Hip circumference | ≥3 years |
| Grip strength | ≥6 years - <85 years |
| Spirometry | ≥35 years - <85 years |
| Cardiovascular disease questionnaire | ≥20 years |
| Intermittent claudication questionnaire | ≥20 years |
| Chronic Obstructive Pulmonary Disease (COPD) questionnaire | ≥35 years |
| Asthma questionnaire | ≥18 years |
| Vitamin D questionnaire | ≥6 months |

**Additional References:**

45 Birch, L. L., J. O. Fisher, K. Grimm-Thomas, C. N. Markey, R. Sawyer, and S. L. Johnson. 2001. 'Confirmatory factor analysis of the Child Feeding Questionnaire: a measure of parental attitudes, beliefs and practices about child feeding and obesity proneness', Appetite, 36: 201-10.

46 Blanton CA, Moshfegh AJ, Baer DJ et al. (2006) The USDA Automated Multiple-Pass Method accurately estimates group total energy and nutrient intake. J Nutr 136, 2594-2599.

47 Bryant, M. J., D. S. Ward, D. Hales, A. Vaughn, R. G. Tabak and J. Stevens (2008). "Reliability and validity of the Healthy Home Survey: a tool to measure factors within homes hypothesized to relate to overweight in children." Int J Behav Nutr Phys Act 5: 23.

48 Burney, P. G., C. Luczynska, S. Chinn, and D. Jarvis. 1994. 'The European Community Respiratory Health Survey', Eur Respir J, 7: 954-60.

49 Buschke, H., Kuslansky, G., Katz, M., Stewart, W. F., Sliwinski, M. J., Eckholdt, H. M. & Lipton, R. B. (1999). Screening for dementia with the memory impairment screen. Neurology, 52, 231-8.

50 Drossman, D. A. 2006. 'The functional gastrointestinal disorders and the Rome III process', Gastroenterology, 130: 1377-90.

51 Economou, M., et al., Suicidal ideation and reported suicide attempts in Greece during the economic crisis. World Psychiatry, 2013. 12(1): p. 53-9.

52 Golan, M. and A. Weizman (1998). "Reliability and validity of the Family Eating and Activity Habits Questionnaire." Eur J Clin Nutr 52(10): 771-777. . Sorensen G et al, Worksite and family education for dietary change: the Treatwell 5-a-Day program. Health Educ Res. 1998 Dec;13(4):577-91.

53 Health Professionals Follow-Up Study Questionnaire (https://sites.sph.harvard.edu/hpfs/hpfs-questionnaires/)

54 National Survey of Children's Health Questionnaire (http://www.childhealthdata.org/learn-about-the-nsch/survey-instruments)

55 Papageorgiou, N., M. Gaga, C. Marossis, C. Reppas, P. Avarlis, M. Kyriakou, S. Tsipra, K. Zeibecoglou, and G. Tracopoulos. 1997. 'Prevalence of asthma and asthma-like symptoms in Athens, Greece', Respir Med, 91: 83-8.

56 Spitzer, R. L., Kroenke, K. & Williams, J. B. (1999). Validation and utility of a self-report version of PRIME-MD: the PHQ primary care study. Primary Care Evaluation of Mental Disorders. Patient Health Questionnaire. JAMA, 282, 1737-44.

57 Wardle, J., C. A. Guthrie, S. Sanderson, and L. Rapoport. 2001. 'Development of the Children's Eating Behaviour Questionnaire', J Child Psychol Psychiatry, 42: 963-70.

58. Holmes TH, Rahe RH. The Social Readjustment Rating Scale. J Psychosom Res. 1967 Aug;11(2):213-8.

59. Karademas, E. C., Tsagaraki, A., & Lambrou, N. (2009). Illness Acceptance, Hospitalization Stress and Subjective Health in a Sample of Chronic Patients Admitted to Hospital. *Journal of Health Psychology*, *14*(8), 1243–1250. <https://doi.org/10.1177/1359105309345169>

# 60. [Subar AF](https://www.ncbi.nlm.nih.gov/pubmed/?term=Subar%20AF%5BAuthor%5D&cauthor=true&cauthor_uid=17000188)^1^, [Dodd KW](https://www.ncbi.nlm.nih.gov/pubmed/?term=Dodd%20KW%5BAuthor%5D&cauthor=true&cauthor_uid=17000188), [Guenther PM](https://www.ncbi.nlm.nih.gov/pubmed/?term=Guenther%20PM%5BAuthor%5D&cauthor=true&cauthor_uid=17000188), [Kipnis V](https://www.ncbi.nlm.nih.gov/pubmed/?term=Kipnis%20V%5BAuthor%5D&cauthor=true&cauthor_uid=17000188), [Midthune D](https://www.ncbi.nlm.nih.gov/pubmed/?term=Midthune%20D%5BAuthor%5D&cauthor=true&cauthor_uid=17000188), [McDowell M](https://www.ncbi.nlm.nih.gov/pubmed/?term=McDowell%20M%5BAuthor%5D&cauthor=true&cauthor_uid=17000188), [Tooze JA](https://www.ncbi.nlm.nih.gov/pubmed/?term=Tooze%20JA%5BAuthor%5D&cauthor=true&cauthor_uid=17000188), [Freedman LS](https://www.ncbi.nlm.nih.gov/pubmed/?term=Freedman%20LS%5BAuthor%5D&cauthor=true&cauthor_uid=17000188), [Krebs-Smith SM](https://www.ncbi.nlm.nih.gov/pubmed/?term=Krebs-Smith%20SM%5BAuthor%5D&cauthor=true&cauthor_uid=17000188). The food propensity questionnaire: concept, development, and validation for use as a covariate in a model to estimate usual food intake.[J Am Diet Assoc.](https://www.ncbi.nlm.nih.gov/pubmed/17000188) 2006 Oct;106(10):1556-63.

61. Willett et al., 2013. Nutritional Epidemiology. Oxford University Press, 2013.
